# Associations of digital neuro-signatures with molecular and neuroimaging measures of brain resilience: The altoida large cohort study

**DOI:** 10.3389/fpsyt.2022.899080

**Published:** 2022-08-09

**Authors:** Azizi A. Seixas, Farid Rajabli, Margaret A. Pericak-Vance, Girardin Jean-Louis, Robbert L. Harms, Ioannis Tarnanas

**Affiliations:** ^1^Department of Psychiatry and Behavioral Sciences, University of Miami Miller School of Medicine, Miami, FL, United States; ^2^John P. Hussman Institute for Human Genomics, University of Miami Miller School of Medicine, Miami, FL, United States; ^3^Altoida Inc., Washington, DC, United States; ^4^Global Brain Health Institute, Trinity College Dublin, Dublin, Ireland

**Keywords:** digital biomarker, dementia, cognitive impairment, Alzheimer's disease, cognitive resilience

## Abstract

**Background:**

Mixed results in the predictive ability of traditional biomarkers to determine cognitive functioning and changes in older adults have led to misdiagnosis and inappropriate treatment plans to address mild cognitive impairment and dementia among older adults. To address this critical gap, the primary goal of the current study is to investigate whether a digital neuro signature (DNS-br) biomarker predicted global cognitive functioning and change over time relative among cognitively impaired and cognitive healthy older adults. The secondary goal is to compare the effect size of the DNS-br biomarker on global cognitive functioning compared to traditional imaging and genomic biomarkers. The tertiary goal is to investigate which demographic and clinical factors predicted DNS-br in cognitively impaired and cognitively healthy older adults.

**Methods:**

We conducted two experiments (Study A and Study B) to assess DNS for brain resilience (DNS-br) against the established FDG-PET brain imaging signature for brain resilience, based on a 10 min digital cognitive assessment tool. Study A was a semi-naturalistic observational study that included 29 participants, age 65+, with mild to moderate mild cognitive impairment and AD diagnosis. Study B was also a semi-naturalistic observational multicenter study which included 496 participants (213 mild cognitive impairment (MCI) and 283 cognitively healthy controls (HC), a total of 525 participants—cognitively healthy (*n* = 283) or diagnosed with MCI (*n* = 213) or AD (*n* = 29).

**Results:**

DNS-br total score and majority of the 11 DNS-br neurocognitive subdomain scores were significantly associated with FDG-PET resilience signature, PIB ratio, cerebral gray matter and white matter volume after adjusting for multiple testing. DNS-br total score predicts cognitive impairment for the 80+ individuals in the Altoida large cohort study. We identified a significant interaction between the DNS-br total score and time, indicating that participants with higher DNS-br total score or FDG-PET in the resilience signature would show less cognitive decline over time.

**Conclusion:**

Our findings highlight that a digital biomarker predicted cognitive functioning and change, which established biomarkers are unable to reliably do. Our findings also offer possible etiologies of MCI and AD, where education did not protect against cognitive decline.

## Introduction

An unprecedented number of people around the world are growing older and living longer. However, an increasing number of them, over the age of 65, will develop cognitive impairment due to late onset Alzheimer's disease (LOAD) (1). Although pathological processes leading to LOAD accumulate over many years prior to disease onset (2) reliably distinguishing declining/poor brain health from normal aging processes remains a challenge. The Lancet Commission's recommendation about the importance of midlife prevention strategies in asymptomatic individuals (3) has spurred several projects to investigate causal models of aging and LOAD risk. Generally, causal models on aging hold the view that aging occurs when there is a loss in either gray and white neuronal matter or other important brain structures, such as the hippocampus (4) and the fornix (5). By definition, such investigations rely on imaging markers and linear mediation analysis (6). However, on numerous occasions the established Alzheimer's disease (AD) biomarkers (amyloid positivity, *APOE4* status) fail to predict cognitive performance in an advanced age (7).

In contrast, cellular, synaptic, and biochemical features of resilient cognition in AD have been positively associated with cognition, representing a “signature” of brain resilience (8, 9). The latest discovery in brain resilience among older adults, from the population-based Mayo Clinic Study of Aging (MCSA), suggests that FDG-PET uptake in the bilateral anterior cingulate cortex and anterior temporal pole was associated with baseline global cognition in cognitively stable 80+ year olds (the resilience signature) and the brain resilience signature provided significant information about global longitudinal cognitive change even when considering amyloid status in both the MCSA and Alzheimer's Disease Neuroimaging Initiative (ADNI) cohorts (10). This signature was significantly related to vascular health and to female gender. Such results supported the predictive role of metabolic changes, and underlined the contribution of preventive factors, specifically vascular risk. Another study found that damage to the support cells (glial cells or astrocytes) may compromise tissue health in the hippocampus and proposed to develop therapies that protect these support cells to fight against the damage aging has on cognitive ability (11). Taken together, such studies would enable predictive algorithms for a brain resilience signature, facilitate identification of brain disease targeting modifiable risk factors, such as vascular health maintenance, and highlight digital biomarker metrics sensitive to the midlife risk of LOAD (12).

However, when focusing on brain resilience being linked to patterns of digital monitoring biomarkers, it is crucial to accurately define both brain health and its determinants through a dynamic trajectory model incorporating antecedent risk factors and also investigate a complex (composite) digital biomarker as a proxy for brain health outcomes (13). With such requirements in mind, two avenues for the validation of digital monitoring biomarkers for brain resilience can be explored: (a) creating a digital biomarker platform based on “digital footprints” for brain function, physical function, social function, protective or risk factors, such as systemic vascular risk and mental health, and/or (b) identifying a unique complex Digital Neuro Signature (14) (DNS)™ that exists as a proxy for brain resilience and disease prevention, such as LOAD (15). To satisfy the first option, a digital brain health platform can be created to assess overall physical health, nutrition, sleep, physical activity, cognitive activity, socialization, and diet recorded *via* smartphones, wearables or other sources of the Internet of Things (IoT) and on the other side inferred casualty with various biological variables (16). Such platforms might contain different classes of digital biomarkers ranging from diagnostic, prognostic, monitoring, pharmacodynamic, predictive to safety and susceptibility digital biomarkers, depending on their unique structure (17). It should be noted that such platforms are still in their infancy and although they can create a metric that is easy for researchers to administer and for recipients to digest, further validation is still needed (18).

In consonance with the second option, a unique DNS monitoring biomarker was evaluated in the current study using remote data acquisition (RDA). This type of biomarker can be extraordinarily useful both in clinical practice and early therapeutic development to determine the quantification of subtle imbalances in the biological network associated with the discordance between FDG-PET measurements from the Alzheimer's disease (AD) regions and resilience processes based on metabolism of the anterior cingulate and anterior temporal lobes. When disease modifying treatments are examined, the DNS monitoring biomarker would serially measure those subtle imbalances, so that changes in the biomarker indicate target engagement and related activity. The ability to measure off-target effects on molecular signatures such as brain metabolism will increasingly come into play, after a recent draft guidance document from the FDA to provide recommendations to sponsors, investigators, and other stakeholders on the use of digital health technologies (DHTs) to acquire data remotely from participants in clinical investigations evaluating medical products (19). Furthermore, to capture the full picture of ongoing brain processes, we describe the longitudinal validation of this monitoring biomarker, based on continuous dynamical interpretation of neuroimaging measurements from the Altoida large cohort study (14). This would enable meaningful monitoring of cognitive change, to collect more precise predictions of therapeutic response through personalized measurements of the pharmacodynamic drug effects coupled with automated date/time stamps.

After we developed the Digital Neuro Signature that corresponds with brain resilience (DNS-br), our goal is to investigate whether the DNS-br biomarker predicted global cognitive functioning and change over time relative to established imaging (amyloid positivity: FDG-PET and PIB ratio) and genetic markers (*APOE4*) of brain health. The second goal of the study is to compare the effect size of the DNS-br biomarker on global cognitive functioning compared to traditional imaging and genomic biomarkers. The third goal of the study is to investigate which demographic and clinical factors predicted DNS-br in cognitively impaired and cognitively healthy older adults.

## Methods

### Study design

We conducted two experiments (Study A and Study B) to assess DNS for brain resilience (DNS-br) against the established FDG-PET brain imaging signature for brain resilience, which emerged from previous studies (10). **Study A** (ClinicalTrials.gov Identifier: NCT02050464) was a semi-naturalistic observational study that included 126 participants, age 65+, with mild to moderate mild cognitive impairment and AD diagnosis recruited in Klinik Hirslanden, Zurich. **Study B** (ClinicalTrials.gov Identifier: NCT02843529) was also a semi-naturalistic observational multicenter study which included 496 participants [213 mild cognitive impairment (MCI) and 283 cognitively healthy controls (HC)], performed in ten European memory clinics and primary care centers, and two primary care community centers in the USA. Thus, a total of 576 participants enrolled in the two studies. These participants were either cognitively healthy (*n* = 303) or diagnosed with MCI (*n* = 253) or AD (*n* = 20). The patients with symptomatic AD pathology gave consent through their study partner. The studies shared similar entry (inclusion/exclusion) criteria and clinical scales, and we characterized the AD biomarkers using the same criteria for the analysis. Both studies were approved by the institutional review board (IRB), i.e., New England IRB in San Diego, USA where the studies were initiated.

In these studies, we included all participants who: (1) had a baseline amyloid and FDG-PET scan, (2) completed the full neuropsychological battery, and (3) were in the Alzheimer's disease cognitive spectrum (cognitively unimpaired, mild cognitive impairment or probable Alzheimer's disease). Thus, in this retrospective observational analysis, our *independent variable* is the testing method, e.g., DNS-br vs. Resilience signature from the FDG-PET and other demographic and imaging variables (elaborated under Materials), and our key *dependent variable* is prediction of global cognitive change in the participants.

### Participants

In both Study A and Study B, we excluded participants with any significant neurologic disease at the recruitment stage, such as Parkinson's disease, Huntington's disease, normal pressure hydrocephalus, brain tumor, progressive supranuclear palsy, seizure disorder, subdural hematoma, multiple sclerosis, or history of significant head trauma followed by persistent neurologic defaults or known structural brain abnormalities. In Study B, further key inclusion criteria were: (1) 55–90 years of age, (2) fluency in English, French, Spanish, Greek, German or Italian, and (3) familiarity with digital devices, including currently possessing and actively using an iPad Pro or iPhone with an at-home Wi-Fi network for the remote assessments. Using these criteria, we first recruited a control group of 200 cognitively healthy individuals from the community that underwent the same procedure at the Global Brain Health Institute (GBHI) at Trinity College, Dublin and 103 cognitively healthy individuals from the Memory Clinics and Primary Care centers.

In recruiting participants with cognitive impairments, the biomarkers (CSF, brain MRI and *APOE* genotype) were used as a criterion and cognitive deficits compatible with MCI diagnosis were found in 253 subjects: 197 from the memory clinics and primary care centers in various countries in Europe and 56 from the community centers in the USA. Seven participants were excluded from the data analysis due to poor data quality. Study A enrolled a total of 120 subjects: 20 healthy controls (HC), 20 mild to moderate Alzheimer's disease (AD) patients, 20 vascular dementia (VAD) patients, 20 fronto-temporal dementia (FTD) patients and 40 subjects with mild cognitive impairment (MCI).

Study B cohort consisted of HC (*n* = 283), and patients with MCI who are at high risk of developing AD within 18–40 months (*n* = 213), assessed every 6 months. Study B enrolled 496 subjects from a total of seven European memory clinics and three primary care centers. The European memory clinics were: 1. Greek Alzheimer's Association and Related Disorders “Ag. Giannis;” 2. “Ag. Eleni” memory clinics in Thessaloniki, Greece of HC (*n* = 3), MCI (*n* = 51) and AD (*n* = 0); 3. the University of Roma La Sapienza memory clinic in Rome of HC (*n* = 2), MCI (*n* = 16) and AD (*n* = 0); 4. IRCCS Centro San Giovanni di Dio Fatebenefratelli memory clinic in Brescia of HC (*n* = 2), MCI (*n* = 14) and AD (*n* = 0) and 5. Neuromed IRCCS memory clinic in Naples, Italy of HC (*n* = 0), MCI (*n* = 1) and AD (*n* = 0); 6. Fundacion Clinic per a la Recerca Biomédica memory clinic in Barcelona, Spain of HC (*n* = 0), MCI (*n* = 35) and AD (*n* = 0); and 7. University of Dublin, Trinity College, St James memory clinic in Dublin, Ireland of HC (*n* = 200), MCI (*n* = 0) and AD (*n* = 0). The three primary care centers from Europe were: BiHELab–Bioinformatics and Human Electrophysiology Lab and affiliated primary physicians' network in Corfu, Greece of HC (*n* = 12), MCI (*n* = 13) and AD (*n* = 0) and two offices from the Practice for Personalized Medicine of the Hirslanden Private Hospital in Switzerland (Zurich and Aarau) of HC (*n* = 12), MCI (*n* = 27) and AD (*n* = 0). Finally, the two primary care community centers in the United States were Scripps Health at La Jolla, California of HC (*n* = 12), MCI (*n* = 35) and AD (*n* = 0) and the Center for Brain Health—The University of Texas at Dallas of HC (*n* = 3), MCI (*n* = 21) and AD (*n* = 0).

The MCI and AD cohorts were included independently on their biomarker status if their diagnosis was consistent with MCI and Alzheimer's dementia diagnosis according to core criteria of NIA-AA revised guidelines (20). Participants were matched on gender and educational level, with no statistically significant difference in cognitive performance between age groups on variables education (*p* = 0.43, Cohen's d = 0.4), or gender (*p* = 0.68, Cohen's d = 0.3). This study monitors a sub-sample of older adults 80+ (*n* = 40) years over time to determine biological and digital biomarker predictors of cognitive resilience, maintaining normal cognition for an average of 40 months, independent of amyloidosis. Demographic characteristics of the cohorts are shown in Tables 1, 2.

**Table 1:** Demographic characteristics of the full sample longitudinal dataset.

	**Study A** **(*n =* 80)**	**Study B (NCT02843529)** **(*n =* 496)**
Follow-up time, years	1.5 (1.0)	2.6 (1.6)
MCI subjects	40	213
Number of MCI subjects progressing to dementia	16 (40%)	100 (47%)
Number of MCI subjects with b-amyloid biomarker progressing to AD dementia	14 (35%)	79 (37%)
MCI subjects progressing to other types of dementia	2 (5%)	21 (10%)
Average Age, years (SD)	77 (10)	67 (8)
Female	44 (56%)	306 (62%)
Male	36 (44%)	190 (38%)
MMSE	26 (2)	27 (2)
Hippocampal volume, cm3	5.3 (1.5)	6.2 (1.2)

*Characteristics of the Study A and Study B participants with available scans and cognitive evaluation are provided (n = 576). Data are n (%) or mean (SD). MMSE, Mini-Mental State Examination*.

**Table 2:** Demographic characteristics of the 80+ sample longitudinal dataset.

**Variables**	**Mean (SD)/%**
Age	81.8 (3.12)
Sex (% male)	52.6%
Education (yrs)	16.45 (2.90)
MMSE (baseline)	26.72 (9.88)
Amyloid positive (%)	54.7%
APOE4 positive (%)	42.3%

*Characteristics of the Study A and Study B participants 80 years and above with available 3T MRI, FDG and AV45 PET scans and cognitive evaluation are provided (n = 40). Of the full sample, 38% were cognitively impaired*.

### Procedure

**Figure 1:**
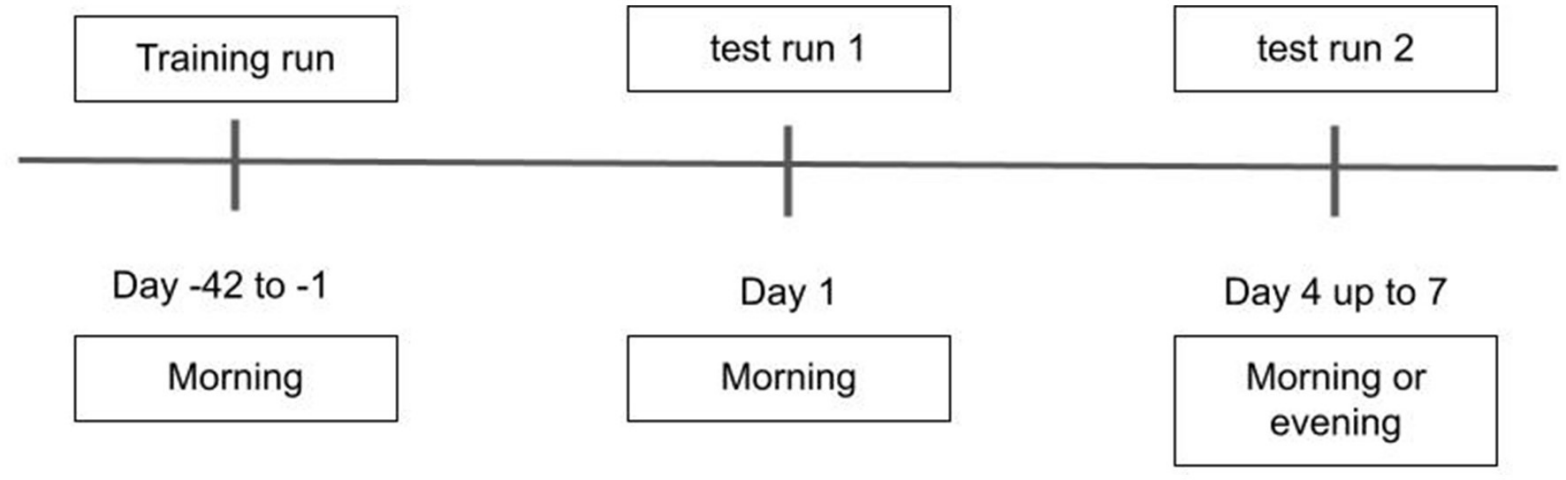
Baseline assessments and timepoints for the administration of the DNS test.

Upon enrollment, all participants gave written informed consent for participation and for reuse of their data. In all groups (HC, MCI and AD), the DNS-br test was administered at the clinic. Half of the participants used DNS-br unsupervised at home on Visit 2 (these measurements were verified against those obtained in the clinic before inclusion in the analysis). An overview of the procedure is represented in Figure 1.

As shown in Figure 1, the first DNS-br total test duration was 20 min including training (10 min training, 2 min break, 8 min measurement). After establishing this baseline, the DNS-br test took an average of 8 min to administer every 6–8 months. The total study duration (including a screening period of up to 42 days) was a maximum of 48 months. Baseline assessments were completed at Training (occurring at 42 to 1 day(s) prior to Day 1), Test Run 1 (Day 1), Test Run 2 (Day 4 up to day 7, morning or evening) (Figure 1). At Test Run 1 assessments were conducted in the morning to avoid the effect of circadian fluctuation in cognitive performance (21). At Test Run 2, a benign cognitive challenge model was implemented to assess the sensitivity of digital endpoints to change. Fatigue and sleep deprivation have been shown to affect performance across a wide range of cognitive domains (22). With that in mind, most baseline assessments in Test Run 2 were conducted in the evening, to produce cognitive fatigue. No napping was allowed prior to the evening assessments, and no caffeine or other stimulants were allowed after 12:00 p.m.

The conventional neuropsychological (NP) assessment took between 120 and 140 min per visit, including breaks. Every 6–8 months, participants were also assessed for their clinical and neuropsychological status with the Mini-Mental State Examination (MMSE) or Montreal Cognitive Assessment (MOCA), and clinically examined if a transition from MCI to dementia (due to AD, or not associated with AD) occurred using a full neuropsychological battery based on the clinical characterization of stages from NIA-AA 2018 (23). Clinical outcomes for MCI/dementia/AD diagnoses were ascertained by investigators blinded to the predictor variables of this study.

Study A participants were tested for a total duration of 18 months between 2013 and 2017, and Study B participants for 40–42 months between 2017 and 2020. Participating memory clinics were in Greece, Italy, Spain, Ireland, Switzerland and the USA.

### Materials

The baseline NP assessments included a comprehensive set of tests: the Wechsler Memory Scale (adjusted for education), MMSE or MOCA, Clinical Dementia Rating (CDR) Memory Box score, and a full neuropsychological (NP) battery including the assessments Digit Span Forward, Digit Span Backward, Trail Making Test A, Trail Making Test B, RAVLT Total, FAQ, GDS, RAVLT A6, RAVLT A7, Benton VRT, Digit Symbol, Block Design, Similarities, and Word and Animal Fluency. These tests, taken together, address 13 cognitive domains.

#### FDG-PET resilience signature

We preprocessed the FDG-PET images using the same pipeline described in the paper from Eider M Arenaza-Urquijo (7). In more detail, a voxel-wise multiple regression analysis was performed in SPM 12 with smoothed and normalized FDG-PET maps and z-global cognition scores for the variables of interest. Following this processing, we used a study-specific gray matter mask to mask for the voxel-wise analysis. We then averaged the segmented and normalized gray matter maps of the study participants and we thresholded to include voxels with a gray matter probability >0.2. The voxel-wise results were considered significant when false discovery rate (FDR) *p* < 0.05 and a cluster extend of *K* > 1,500 mm3. Reference anatomical gray matter labels were determined using the Mayo Clinic Adult Lifespan Template (MCALT).Then, we used normalized FDG-PET maps and extracted a single FDG-PET value from the ‘resilience signature' described in the paper above. We also extracted a single FDG-PET value from the same areas described in the paper (see “global FDG-PET ratio measure” section in methods) that was used as an FDG based AD biomarker.

#### PET AD imaging biomarkers

We used available PET tracer (11) C-labeled Pittsburgh Compound-B ((11) C-PIB) and AV45-PET SUVR values as a measure of global cortical amyloid retention. The amyloid status from PET was defined based on a cut-off of 1.11 (24).

#### Acquisition and measurement of MRI variables

Participants were imaged by a Siemens 1.5T field strength machine (Siemens Medical, Erlangen, Germany) with a 3-dimensional T1-weighted coronal spoiled gradient-recalled echo (SPGR) sequence. Segmentation of brain structural MRI was performed by semi-automated procedures, for example, gray matter, white matter, and CSF segmentation were performed using an Expectation-Maximization (EM) algorithm after skull-stripping, and Intensity Inhomogeneity Correction. Hippocampus was segmented by the multiatlas hippocampal segmentation algorithm described elsewhere (25). The primary MRI measure was total cerebral brain volume (TCBV) with cerebral white matter volume, cerebral gray matter volume, hippocampal volume, and WMH volume and white matter hyperintensity as secondary measures. The large WMH volume (WMH-Large) was defined as those with more than one standard deviation higher than the age-specific mean values, using the Fazekas scale.

Additionally, we collected fluid AD biomarkers, consisting of β-amyloid and *p*-tau and total tau protein *cerebrospinal fluid* (CSF) levels, brain MRI and *APOE* genotype. To ensure a finer understanding of the type of cognitive impairment, classification in the diagnostic clusters of MCI and dementia due to AD (aMCI and ADD), or MCI and dementia not associated with AD (naMCI and nADD), were performed based on the β-amyloid and tau protein CSF levels biomarker.

#### Digital neuo signature brain resilience (DNS-Br) biomarker

For this work, we analyzed data from Altoida's DNS application which collects digital biomarkers for neurocognitive function measurement and progression tracking for AD (26). The Altoida DNS captures 793 active digital biomarkers, such as reaction time, speed, attention- and memory-based assessments, as well as every single device sensor input (or lack thereof) through accelerometer, gyroscope, magnetoscope, camera, microphone, and touch screen. We piloted Altoida DNS in an independent pilot study with a sample of young, healthy controls across all Altoida cognitive domains, and found that test-retest variability was 0.156% (27). Such low variability shows excellent internal validity of the Altoida DNS test and corroborates the represent ability and stability of its measures over time.

**Figure 2:**
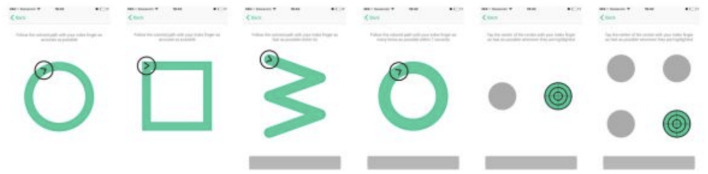
The motoric functioning tasks in the Altoida DNS test. These are executed one after another. Using their index finger of their dominant hand, from left to right, the task is to (1) draw a circle, (2) draw a square, (3) draw a rotated W shape within 7 s, (4) draw as many circles as possible within 7 s, (5) tap the highlighted buttons (left, right, left, right, etc.) (6) tap the highlighted button as fast as possible, the buttons highlight at random.

**Figure 3:**
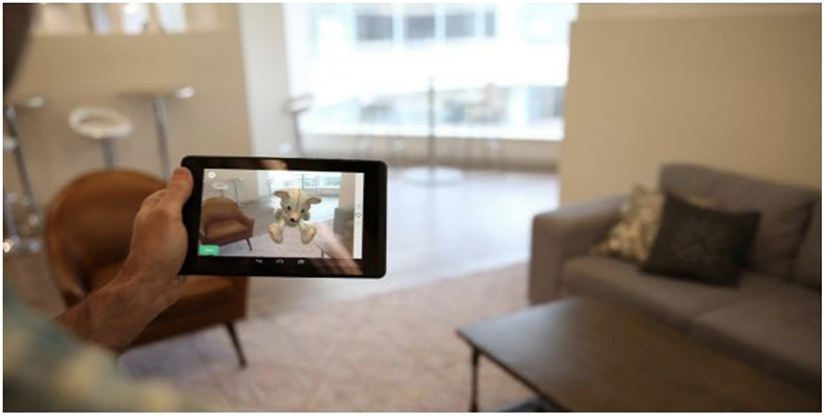
Illustration of the Augmented Reality (AR) task in the Altoida DNS test. During the AR test, the subject is asked to place and find three virtual objects in the room. To do so, the subject is required to walk around the room holding a tablet or smartphone device in front of him/her. While doing so, the camera of the device records the environment and displays it back to the user on the screen, augmented with virtual objects (in this illustration, a teddy bear). The user needs to place the objects on flat surfaces and later recall their position by walking back to that location.

While holding a tablet or smartphone device, the subject is asked to perform a series of motor functioning tasks and two Augmented Reality (AR) tasks. In the motor functioning tasks, the subject is required to draw shapes and tap on the (touch) screen using the finger of their dominant hand (see Figure 2 for an illustration of all the motor functioning tasks). In one of the AR tasks, the subject is asked to place three virtual objects in a small space (~3 x 3 or 2 x 4 m) and afterward find them again. The AR task is performed by navigating around the space with the tablet or smartphone in both hands (see Figure 3). During these tasks, the handheld device collects telemetry and touch data from the built-in sensors, enabling profiling of hand micro-movements, screen touch pressures, walking speed, navigation trajectory, cognitive processing speed, and additional proprietary inputs.

A single test session using Altoida's application consists of two batches of motor tasks and two AR tasks. After a subject completes all tasks, the recorded digital biomarker data from the onboard electronics sensors is bundled and securely and anonymously uploaded to a server for further processing. Provided the data of multiple subjects, machine learning can be used to detect patterns. In previous work, machine learning was either used to classify subjects as healthy or at risk of AD (26). In this work, we examined DNS signatures from our validated dataset for AD to see if they demonstrated preclinical markers that predict cognitive resilience in the older adult individuals 80+, who could maintain normal cognition for an average of 40 months, independently from amyloidosis. Such markers are expressed by the capacity of the DNS measurement one-time results to inform a novel “digital” brain resilience DNS signature.

#### DNS-Br machine learning

We extracted 793 digital biomarker features from the onboard electronics sensors describing various cognitive, functional, and physiological characteristics of each subject. These features include response times, eye-hand coordination precision, fluctuations in the telemetry (accelerometer and gyroscope) data, Fourier analysis of the telemetry data, step detection, and additional proprietary data. Based on the digital biomarker feature data from a selection of healthy subjects, we trained a DNS-br match classifier to distinguish “high” brain resilience individuals ≥ 80 years old that would maintain normal cognition during follow-ups for an average of 40 months. We used the XGBoost algorithm with DNS preclinical markers that predict cognitively stable older adults 80+ as the target variable for the classification.

##### DNS-Br: Performance evaluation

We applied stratified 5-fold grouped cross-validation to estimate the generalization performance of the DNS-br classifier. We grouped data points by subject to ensure that multiple data points of a single subject were all in the same fold (either training or testing), preventing learning bias. For our classifier, we measured accuracy and precision averaged over the five cross-validation testing folds. To assess the classifier's performance on different age groups, we trained nine additional classifiers (10 in total), each using different random subsets of the data. This machine learning (ML) classifier is a re-purposed version of the classifier described in a previous study (24) that had an excellent performance (ROC-AUC 0.91) when examining individuals that convert to dementia, independently of their Aβ biomarker value, compared to healthy subjects that remain stable over 3 years (**Table 4**, column 1).

##### DNS-Br: Model explainability

We used the Shapley Additive exPlanations (SHAP) (28) method to better understand the predictions made by the DNS brain resilience classifier. The method for obtaining Shapley values emerges from the context where “n” players participate collectively obtaining a reward “p” which is intended to be fairly distributed at each one of the “n” players according to the individual contribution, such a contribution is a Shapley value. In simple words, a Shapley value is the average marginal contribution of an instance of a feature among all possible coalitions. The SHAP method allocates to each feature of a classifier a game-theoretical value representing the contribution of that feature toward the classification targets. This is used for the interpretation of predictions of ML models through Shapely values. The key idea of SHAP is to calculate the Shapley values for each feature of the sample to be interpreted, where each Shapley value represents the impact that the feature to which it is associated, generates in the prediction. The sign of the SHAP values indicates the direction of the contribution, and the magnitude of the SHAP value indicates the importance. For our classifier, negative SHAP values contribute to classifying as non-resilient, positive numbers toward resilient. SHAP values have an additive property meaning they can be summed together to provide the feature contribution of a group of features.

### Statistical analyses

We conducted statistical analyses similar to the manuscript from Arenaza-Urquijo (10). We first fitted a multiple regression model for predicting baseline cognition (gold standard NP assessments) including demographic variables (age, sex, education), APOE4 status, DNS-br signature, and imaging variables (“resilience signature,” amyloid burden and FDG-PET from the AD signature). The objective of these analyses was to evaluate whether DNS-br associated with the FDG-PET uptake in the “resilience signature” predicted cognition over and above AD biomarkers. Linear regression models were used to assess the associations of DNS-br scores with FDG-PET uptake measures and imaging variables, adjusting for age, sex, and education. In the sensitivity analysis, the models were additionally adjusted for vascular risk factors including hypertension, diabetes, smoking and prevalent atrial fibrillation. We used bonferroni correction to adjust for multiple testing in the linear regression models and the significant associations were claimed if *P* < 0.05/N, where N was the number of tests performed.

Second, we evaluated whether DNS-br was associated with the FDG-PET uptake in the “resilience signature,” in order to predict longitudinal change in global cognition taking into account amyloid status. To this aim, we fitted a linear mixed effect model with global cognition as dependent variable, and time (age at visit), amyloid status, sex, years of education, FDG-PET, APOE4 status as fixed effects (predictors). The model included all main effects as well as interactions with time, including our interactions of interest: DNS-br^*^time. Finally, we fitted a linear mixed effects model for the predictive value of different risk factors in the DNS-br signature in stable vs. cognitively impaired individuals. Random effects for intercept and slopes were included.

## Results

As shown in Table 3, DNS-br total score and majority of the 11 DNS-br neurocognitive subdomain scores were significantly associated with FDG-PET resilience signature, PIB ratio, cerebral gray matter and white matter volume, after adjusting for multiple testing. Also, the effect size of DNS-br for FDG-PET resilience signature and PIB ratio were higher, as compared to Cerebral white matter volume and Cerebral gray matter volume. In general, greater DNS-br score was associated with both the FDG-PET resilience signature and PIB ratio.

**Table 3:** Association of DNS-br scores with FDG-PET resilience signature, PIB ratio, and cerebral white matter and gray matter volumes.

**DNS-br**	**FDG-PET resilience signature**			**PIB ratio**			**Cerebral white matter volume**			**Cerebral gray matter volume**		
	**Effect size**	**Standard error**	***P*-value ^a^**	**Effect size**	**Standard error**	***P*-value ^a^**	**Effect size**	**Standard error**	***P*-value ^a^**	**Effect size**	**Standard error**	***P*-value ^a^**
DNS-br total	9.1 ×10^−2^	2.5 ×10^−2^	**4.9** × **10**^−4^	9.2 ×10^−2^	2.2 ×10^−2^	**4.8** × **10**^−4^	4.5 ×10^−2^	2.5 ×10^−2^	2.4 ×10^−2^	4.3 ×10^−2^	2.4 ×10^−2^	2.0 ×10^−2^
DNS-br perceptual motor coordination	8.9 ×10^−2^	2.5 ×10^−2^	**5.1** × **10**^−4^	8.8 ×10^−2^	2.2 ×10^−2^	**7.0** × **10**^−4^	5.2 ×10^−2^	2.5 ×10^−2^	**7.3** × **10**^−4^	5.8 ×10^−2^	2.4 ×10^−2^	**3.9** × **10**^−^^4^
DNS-br complex attention	7.2 ×10^−2^	2.5 ×10^−2^	**4.0** × **10**^−4^	7.6 ×10^−2^	2.2 ×10^−2^	**7.1** × **10**^−4^	4.8 ×10^−2^	2.5 ×10^−2^	4.6 ×10^−3^	4.9 ×10^−2^	2.4 ×10^−2^	7.7 ×10^−1^
DNS-br cognitive processing speed	8.6 ×10^−2^	2.5 ×10^−2^	**4.2** × **10**^−4^	7.9 ×10^−2^	2.2 ×10^−2^	**5.6** × **10**^−4^	5.4 ×10^−2^	2.6 ×10^−2^	3.8 ×10^−2^	5.6 ×10^−2^	2.4 ×10^−2^	1.4 ×10^−2^
DNS-br inhibition	8.9 ×10^−2^	2.5 ×10^−2^	**5.0** × **10**^−4^	8.8 ×10^−2^	2.2 ×10^−2^	**6.3** × **10**^−4^	5.7 ×10^−2^	2.6 ×10^−2^	**6.9** × **10**^−5^	5.6 ×10^−2^	2.4 ×10^−2^	4.7 ×10^−2^
DNS-br flexibility	9.0 ×10^−2^	2.5 ×10^−2^	**5.6** × **10**^−4^	8.6 ×10^−2^	2.2 ×10^−2^	**5.5** × **10**^−4^	6.2 ×10^−2^	2.6 ×10^−2^	**7.2** × **10**^−5^	5.4 ×10^−2^	2.4 ×10^−2^	**6.3** × **10**^−4^
DNS-br visual perception	8.0 ×10^−2^	2.5 ×10^−2^	**2.3** × **10**^−3^	8.4 ×10^−2^	2.2 ×10^−2^	**2.6** × **10**^−3^	4.5 ×10^−2^	2.5 ×10^−2^	2.3 ×10^−1^	4.1 ×10^−2^	2.4 ×10^−2^	1.7 ×10^−1^
DNS-br planning	7.8 ×10^−2^	2.5 ×10^−2^	**7.3** × **10**^−4^	8.4 ×10^−2^	2.2 ×10^−2^	2.4 ×10^−2^	4.1 ×10^−2^	2.6 ×10^−2^	2.1 ×10^−1^	4.6 ×10^−2^	2.4 ×10^−2^	1.4 ×10^−1^
DNS-br prospective memory	8.5 ×10^−2^	2.5 ×10^−2^	3.2 ×10^−3^	7.6 ×10^−2^	2.2 ×10^−2^	6.2 ×10^−2^	4.6 ×10^−2^	2.5 ×10^−2^	5.3 ×10^−2^	4.7 ×10^−2^	2.4 ×10^−2^	6.3 ×10^−2^
DNS-br eye movement	6.9 ×10^−2^	2.5 ×10^−2^	3.7 ×10^−3^	7.1 ×10^−2^	2.2 ×10^−2^	4.4 ×10^−2^	5.6 ×10^−2^	2.5 ×10^−2^	3.5 ×10^−1^	5.4 ×10^−2^	2.4 ×10^−2^	2.4 ×10^−1^
DNS-br spatial memory	8.9 ×10^−2^	2.5 ×10^−2^	3.0 ×10^−3^	8.7 ×10^−2^	2.2 ×10^−2^	3.3 ×10^−3^	4.8 ×10^−2^	2.5 ×10^−2^	2.7 ×10^−1^	5.3 ×10^−2^	2.4 ×10^−2^	3.1 ×10^−1^
DNS-br speech and articulation	5.6 ×10^−2^	2.5 ×10^−2^	2.7 ×10^−2^	5.4 ×10^−2^	2.2 ×10^−2^	7.1 ×10^−2^	3.3 ×10^−2^	2.5 ×10^−2^	4.0 ×10^−1^	3.3 ×10^−2^	2.5 ×10^−2^	4.0 ×10^−1^

*This model was adjusted for age, sex, and education. The FDG-PET uptake in the resilience signature was considered low (<5th percentile), average (50th percentile) and high (>95th percentile). PIB ratio was the amyloid status in baseline. The MRI measures were the percent of these volumes over the total cerebral cranial volume (TCV) above the tentorium.*

a *Bonferroni correction was used to adjust for multiple testing. Significant associations were claimed if p <0.05/2 (2.9 ×10^−3^) and indicated in bold, where 2 was the number of DNS-br performed (biannual)*.

In Table 4, we investigated whether the DNS-br total score and the 11 DNS-br neurocognitive subdomains were associated with FDG-PET resilience signature, PIB ratio, cerebral gray matter and white matter volume, adjusting for vascular risk factors. FDG-PET resilience signature was significantly associated with DNS-br total score and all subdomains except for DNS Speech and articulation (all with *p* < 2.9 ×10^−3^). PIB ratio was significantly associated with DNS-br total and all DNS-br subdomain scores except for DNS-br Speech and Articulation and DNS-br Eye Movement. Cerebral white matter volume and Cerebral gray matter volume and DNS-br were not associated with DNS-br-total score, but were associated with a few DNS-br subdomains. No differences were found between the clinic administered version of DNS-br and the unsupervised home administration.

**Table 4:** Association of DNS-br scores with FDG-PET resilience signature, PIB ratio, and cerebral white matter and gray matter volumes after additionally adjusting for vascular risk factors.

**DNS-br**	**FDG-PET resilience signature**			**PIB ratio**			**Cerebral white matter volume**			**Cerebral gray matter volume**		
	**Effect size**	**Standard error**	***P*-value ^a^**	**Effect size**	**Standard error**	***P*-value ^a^**	**Effect size**	**Standard error**	***P*-value ^a^**	**Effect size**	**Standard error**	***P*-value ^a^**
DNS-br total	9.5 ×10^−2^	2.5 ×10^−2^	**6.8** × **10**^−5^	10.1 ×10^−2^	2.2 ×10^−2^	**2.5** × **10**^−4^	5.6 ×10^−2^	2.5 ×10^−2^	5.3 ×10^−2^	5.3 ×10^−2^	2.4 ×10^−2^	9.5 ×10^−2^
DNS-br perceptual motor coordination	9.9 ×10^−2^	2.5 ×10^−2^	**7.1** × **10**^−5^	9.4 ×10^−2^	2.2 ×10^−2^	**5.9** × **10**^−4^	6.6 ×10^−2^	2.5 ×10^−2^	**7.3** × **10**^−4^	6.9 ×10^−2^	2.5 ×10^−2^	**8.2** × **10**^−^^4^
DNS-br complex attention	8.1 ×10^−2^	2.5 ×10^−2^	**5.1** × **10**^−4^	7.9 ×10^−2^	2.2 ×10^−2^	**3.8** × **10**^−4^	5.3 ×10^−2^	2.5 ×10^−2^	3.5 ×10^−3^	5.3 ×10^−2^	2.4 ×10^−2^	6.2 ×10^−2^
DNS-br cognitive processing speed	8.9 ×10^−2^	2.5 ×10^−2^	**4.2** × **10**^−4^	8.2 ×10^−2^	2.2 ×10^−2^	**2.7** × **10**^−4^	6.1 ×10^−2^	2.6 ×10^−2^	9.1 ×10^−2^	6.3 ×10^−2^	2.5 ×10^−2^	4.5 ×10^−2^
DNS-br inhibition	9.9 ×10^−2^	2.4 ×10^−2^	**8.2** × **10**^−5^	9.8 ×10^−2^	2.2 ×10^−2^	**3.3** × **10**^−4^	7.3 ×10^−2^	2.6 ×10^−2^	**6.9** × **10**^−5^	6.6 ×10^−2^	2.4 ×10^−2^	7.4 ×10^−2^
DNS-br flexibility	9.6 ×10^−2^	2.5 ×10^−2^	**5.5** × **10**^−5^	9.3 ×10^−2^	2.2 ×10^−2^	**9.4** × **10**^−4^	7.9 ×10^−2^	2.6 ×10^−2^	**7.2** × **10**^−5^	8.4 ×10^−2^	2.4 ×10^−2^	**8.8** × **10**^−5^
DNS-br visual perception	8.7 ×10^−2^	2.5 ×10^−2^	**4.3** × **10**^−5^	8.4 ×10^−2^	2.2 ×10^−2^	**5.7** × **10**^−4^	5.7 ×10^−2^	2.5 ×10^−2^	4.8 ×10^−1^	5.2 ×10^−2^	2.4 ×10^−2^	9.7 ×10^−2^
DNS-br planning	7.9 ×10^−2^	2.6 ×10^−2^	**6.9** × **10**^−4^	8.8 ×10^−2^	2.2 ×10^−2^	4.2 ×10^−3^	5.2 ×10^−2^	2.6 ×10^−2^	4.1 ×10^−2^	5.4 ×10^−2^	2.4 ×10^−2^	3.2 ×10^−2^
DNS-br prospective memory	9.5 ×10^−2^	2.5 ×10^−2^	**8.3** × **10**^−5^	8.6 ×10^−2^	2.2 ×10^−2^	**4.8** × **10**^−4^	5.6 ×10^−2^	2.5 ×10^−2^	3.9 ×10^−2^	5.5 ×10^−2^	2.4 ×10^−2^	9.5 ×10^−2^
DNS-br eye movement	7.4 ×10^−2^	2.5 ×10^−2^	**6.7** × **10**^−4^	7.6 ×10^−2^	2.2 ×10^−2^	5.4 ×10^−3^	5.8 ×10^−2^	2.5 ×10^−2^	**6.3** × **10**^−4^	6.4 ×10^−2^	2.3 ×10^−2^	**8.3** × **10**^−5^
DNS-br spatial memory	8.9 ×10^−2^	2.5 ×10^−2^	**8.1** × **10**^−5^	8.9 ×10^−2^	2.2 ×10^−2^	**2.8** × **10**^−5^	5.3 ×10^−2^	2.5 ×10^−2^	**5.4** × **10**^−5^	5.6 ×10^−2^	2.4 ×10^−2^	5.9 ×10^−2^
DNS-br speech and articulation	7.9 ×10^−2^	2.5 ×10^−2^	3.8 ×10^−3^	6.2 ×10^−2^	2.2 ×10^−2^	5.3 ×10^−3^	5.7 ×10^−2^	2.5 ×10^−2^	8.2 ×10^−2^	5.5 ×10^−2^	2.4 ×10^−2^	9.0 ×10^−2^

*This model was adjusted for age, sex, and education. The FDG-PET uptake in the resilience signature was considered low (<5th percentile), average (50th percentile) and high (>95th percentile). PIB ratio was the amyloid status in baseline. The MRI measures were the percent of these volumes over the total cerebral cranial volume (TCV) above the tentorium.*

a *Bonferroni correction was used to adjust for multiple testing. Significant associations were claimed if p <0.05/2 (2.9 ×10^−3^) and indicated in bold, where 2 was the number of DNS-br performed (biannual)*.

To understand predictors of global cognitive functioning among individuals 80 years and older, we performed linear regression analyses and found that sex, education (yrs), APOE4 status, DNS-br Resilience, sig FDG-PET, and AV45-PET ratio significantly predicted global cognitive functioning. Specifically, sex, APOE4 status, and AV45-PET ratio positively predicted global cognitive functioning. While, education, DNS-br resilience and sig FDG-PET negatively predicted global cognitive functioning (See Table 5). To investigate which factors predicted cognitive changes among cognitive stable and impaired individuals, we performed a mixed effect model looking at the independent and interaction predictors (See Table 6). Sex was the only factor that was significantly associated with cognitive change. Although sex was positively associated with changes in cognitive status among cognitive normal and impaired individuals, its interaction with age was negatively associated. Additionally, interactions of age^*^DNS-br total and age^*^FDG-PET were negatively associated with cognitive change. Conversely, the interaction between age and AV45-PET was positively associated (See Table 6). Lastly, we investigated which factors predicted DNS-br in cognitively stable and impaired individuals. Among cognitively stable individuals, education, hypertension, diabetes, smoking, and atrial fibrillation predicted DNS-br total (See Table 7). While for cognitively impaired individuals, age, education, hypertension, diabetes, and atrial fibrillation predicted DNS-br total (See Table 7).

**Table 5:** Results of the linear multiple regression models to predict impairment of your global cognition in the 80+ participants Beta coefficients, confidence intervals (CI) for unstandardized Betas and *p*-values are provided.

	**R2**	**Beta**	**95% CI**	***p*-value**
**Model**	0.45			
Intercept		76.80	42.91–91.78	<0.001
**Demographic variables**				
Age		−1.63	−1.42–0.196	0.238
Sex		3.42	0.90–5.13	0.005
Education (yrs)		−0.655	−0.81–0.17	0.003
APOE4 status		3.29	0.32–4.33	0.030
**Digital response biomarkers**				
DNS-br		−27.82	−47.13 −−17.57	<0.001
**Imaging variables**				
Resilience sig FDG-PET		−33.67	−44.90 −−18.34	<0.001
AD sig FDG-PET		8.511	7.38–17.03	0.109
AV45-PET ratio		10.18	7.87–14.22	<0.001

*AD sig, Alzheimer's disease signature. Significant findings: p-value <0.05, p <0.01, p <0.001 significant*.

**Table 6:** Results of the mixed effect model to predict cognitive change in the full sample of 80+ participants (stable and impaired).

	**Beta**	**95% CI**	***p*-value**
Intercept	−86.18	−255.32–62.72	0.001
Time (age at visit)	2.51	−0.085–4.910	0.060
Sex	29.23	5.80–59.11	0.01
Education (yrs)	−3.75	−6.22–2.67	0.55
APOE4 status	8.89	−29.71–41.86	0.51
DNS-br total	91.23	−35.40–211.68	0.08
“Resilience sig” FDG-PET	74.69	−34.23–198.09	0.21
AV45-PET status	−59.77	−76.43 −−36.87	<0.001
Sex*time	−0.29	−0.66 −−0.09	0.02
Education (yrs) *time	0.05	−0.03–0.08	0.42
APOE4 status *time	−1.07	−12.19–26.68	0.71
DNS-br*time	−7.38	−12.78 −−0.23	<0.001
Resilience sig FDG-PET *time	−1.45	−2.56 −−0.09	0.043
AV45-PET status*time	0.99	0.64–2.31	<0.001

*Beta coefficients, confidence intervals (CI) for unstandardized Betas and p values are provided. AD sig: Alzheimer's disease signature. Cognitive change was calculated based on the clinical characterization of stages using the NIA-AA Alzheimer's Disease Framework 2018 (24). Significant findings: p-value <0.05, p <0.01, p <0.001 significant*.

**Table 7:** Assessment of the predictive value of different risk factors in the DNS-br signature.

	**Cognitively stable 80**+ **(*****N*** = **32)**				**Cognitively impaired 80**+ **(*****N*** = **207)**			
	**R2**	**Beta**	**CI**	***p*-value**	**R2**	**Beta**	**CI**	***p*-value**
Model	0.28				0.33	
Intercept^a^		−1.68	−7.17–2.55	0.429		−0.983	−5.19–2.14	0.573
**Demographic variables**								
Age^a^		−0.05	−0.08–0.002	0.062		0.072	−0.09 −−0.045	<0.001
Sex^a^		−0.16	−0.45–0.09	0.260		1.97	2.28 − –0.07	0.371
Education (yrs) ^a^		0.09	0.05–0.15	<0.001		−0.100	−0.06 −−0.14	<0.001
APOE4 status ^a^		−0.14	−0.50–0.26	0.273		1.93	2.29–0.32	0.226
**Risk factor variables**								
Hypertension ^b^		−2.71	−5.31 −−1.04	<0.001		2.61	1.12–4.32	<0.001
Diabetes ^b^		−3.39	−5.09 −−1.68	0.010		4.17	1.08–6.39	0.005
Smoking ^b^		−2.19	−4.52 −−1.9	0.005		2.09	1.51–4.98	0.121
Prevalent atrial fibrillation ^b^		−4.05	−7.26 −−1.2	<0.001		5.29	1.49–7.08	<0.001

*The table below shows the unadjusted R square, beta coefficients, confident intervals (CI).*

a *: Unstandardized Beta coefficient;*

b *: Standardized Beta coefficients. Significant findings: p-value <0.05, p <0.01, p <0.001 significant*.

**Figure 4:**
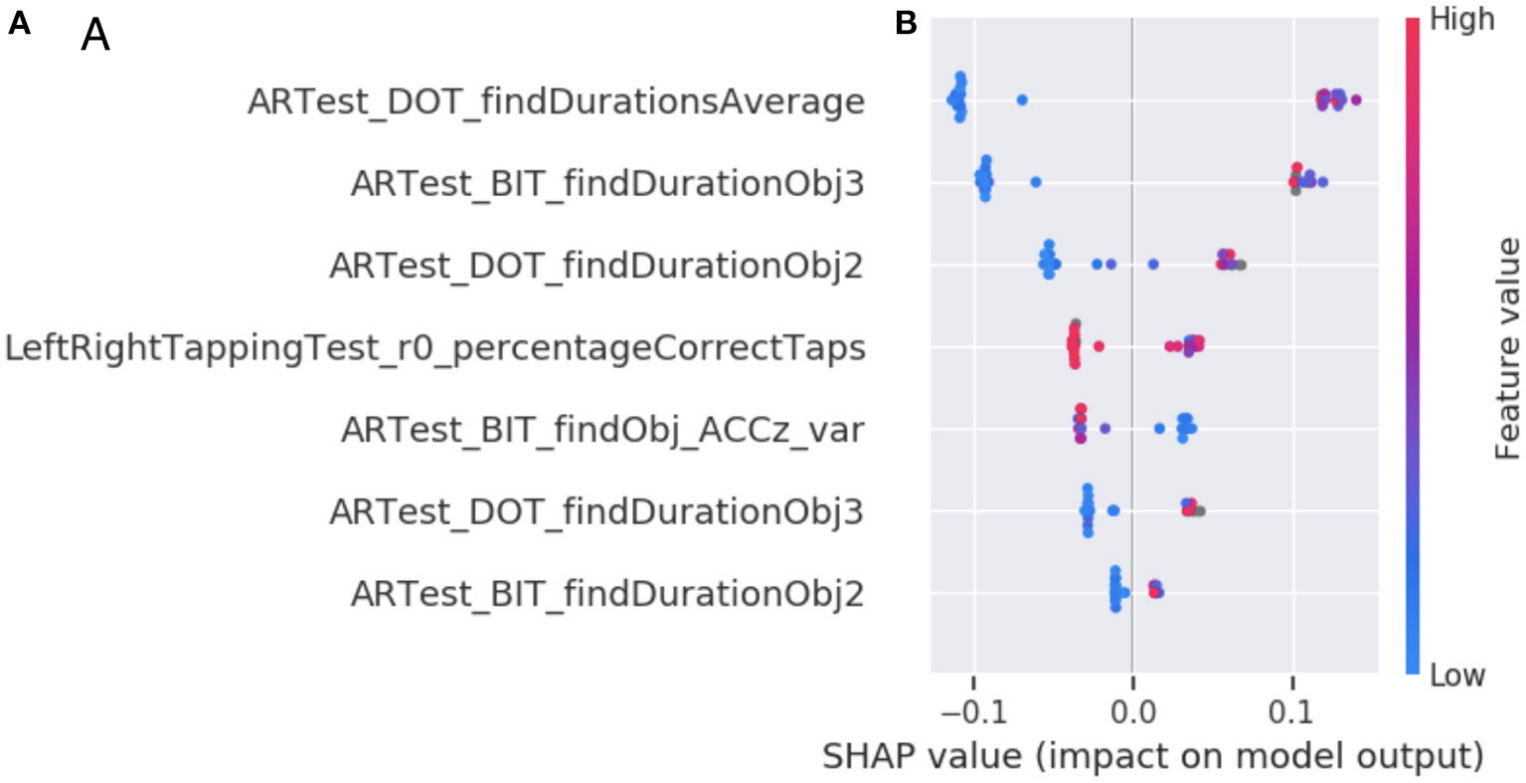
Feature importance of the DNS-br classifier. **(A)** The top seven digital biomarker groups according to the SHAP method. Each bar represents the summed SHAP value of the features in that feature group. **(B)** A feature value SHAP distribution plot for the top seven contributing features. Subject specific SHAP values were computed for each datapoint in the classifier training data. For each feature, we then plot for each datapoint a dot with the feature value of that datapoint, with the dot color coded by the relative feature value. On the left side we observe each feature ordered according to its importance. The perimeter feature being the most important and texture feature being the least important. The color represents the values that each feature can take, red for high values and blue for low values. Therefore, for the feature DOT average find durations, if the values are high (red) the Shapley values will be low and consequently it will be pushing toward class 0 (or high brain resilience and remaining stable at 80+), otherwise, when the values are low (blue) the Shapley values they will be high that consequently they will be pushing toward class 1 (or low brain resilience and potential cognitive change at 80+). The position of each dot on the SHAP value x-axis represents the magnitude and the direction of the contribution of that specific feature value of that specific datapoint toward classifying as resilient (-1) or non-resilient (+1). Acronyms in the plots are Augmented Reality (AR), Day out Task test (DOT), Back in Time test (BIT), Accelerometer (ACC), variance (var), first part of a single test (1st) or second part of a single test (2nd).

Lastly, based on SHAP, the primary contributing group of active digital biomarkers is AR object finding durations. This group consists of the participant fine-movements while trying to find a virtual object in the AR test (micro-movements). The second most important group of digital biomarker features is the tapping variance. The tapping variance can be interpreted as coarse-scale hand motion micro-movement (motor feature). The third and fourth most essential features are again micro-movements during object finding, as they account for cognitive decline over time accounting for amyloid status (Figure 4).

## Discussion

The primary goal of the current study was to investigate whether a digital neurosignature (DNS) biomarker of brain resilience, (DNS-br), predicted global cognitive functioning and cognitive change in cognitively healthy and impaired individuals 80+ years old. Specifically, we investigated associations between DNS-br total (and its 11 subdomains) and established imaging (amyloid positivity: FDG-PET and PIB ratio) and biological markers (APOE4) of brain health and cognitive resilience. The secondary goal was to compare the effect size of the DNS-br biomarker on global cognitive functioning compared to traditional imaging and genomic biomarkers. The third goal was to investigate which demographic and clinical factors predicted DNS-br in cognitively impaired and cognitively stable older adults. Our findings provide very promising results that DNS-br (a digital biomarker) may be a comparable or greater predictor of global cognitive functioning, as traditional imaging biomarkers and biological markers of brain health and resilience. To investigate these three research questions, we monitored the sub-sample of older adults 80+ years over time to determine biological and digital biomarker predictors of cognitive resilience, maintaining normal cognition for an average of 40 months, independent of amyloidosis.

These findings have both significant clinical and public health implications. Clinically, our findings suggest possible common and unique risk and protective profiles of brain health and resilience among cognitively stable and impaired older adults. These profiles may manifest in unique symptom presentation and progression of cognitive decline, which in turn may provide insight about which types of treatments (pharmacological, cognitive, or behavioral treatments) may be used to prevent or manage cognitive decline, a vision of precision neurology. The public health implications of our findings are also noteworthy as the successful use of a digital health technology, like Altoida, may increase access to early screening, diagnosis, treatment and management of dementia symptoms, thus curtailing the burden of dementia.

### Digital biomarker and brain health, pathology and resilience

Although established dementia biomarkers, like FDG-PET, PIB ratio, cortical thickness, beta amyloid and APOE4, are widely used as risk markers for cognitive impairment, decline, and dementia, contradictory evidence that they do not predict global or future cognitive functioning, change, or decline, call into question their robustness in predicting dementia among older adults (10). Recent studies highlight that these established biomarkers are better predictors of baseline cognition as opposed to future cognition or changes in cognition (10). These discrepancies in traditional dementia biomarkers undermine their clinical and public health value and provides insight on why older adults (80+ years) who present with positive biological signs of dementia and no clinically significant cognitive impairment. It is likely that older adults with imaging and genetic risk markers (APOE E4 carrier) may be cognitively stable. This calls into question whether interventions are needed for someone with biological risk but no cognitive and functional impairment. Established biomarkers may not be the most sensitive predictors of dynamic changes in cognition, and suggest instead a digital biomarker may be more sensitive.

Therefore, better instruments are needed to: assess probability of long-term (and not cross-sectional or short term decline) cognitive decline, predict changes in cognitive status, and differentiate biological risk and functional cognitive impairment and decline, as we cannot rely on traditional biomarkers. Our study fills a critical void in the literature by suggesting that a digital biomarker for functional brain resilience, DNS-br, might be a better predictor of cognitive impairment and decline and may help us to identify and distinguish individuals with a biomarker risk and cognitive impairment and those with a biomarker risk and who are cognitively healthy.

In the current study, we establish that a novel Digital Neuro Signature, in the context of functional brain resilience, which captures over 800 active digital biomarkers through accelerometer, gyroscope, magnetoscope, camera, microphone, and touch screen, is a superior predictor of global cognitive functioning and changes over time compared to traditional neuropsychological assessments, for asymptomatic at-risk older adults. For example, in Table 3, DNS-br total score was significantly associated with several brain health markers such as: FDG-PET resilience signature and PIB ratio, but not cerebral white matter volume, and cerebral gray matter volume. Even after adjusting for vascular risk factors, the relationships remained the same albeit the magnitudes of the effect size increased.

However, in more granular analyses, DNS-br perceptual motor coordination and flexibility were associated with PET resilience signature and PIB ratio, cerebral white matter volume, and cerebral gray matter volume. After adjusting for vascular risk factors, these relationships remained the same and the effect sizes increased. DNS-br inhibition was associated with FDG-PET resilience signature, PIB ratio, and cerebral white matter volume for unadjusted and adjusted models (vascular risk). Effect sizes in the adjusted model were greater compared to the unadjusted model.

DNS-br complex attention, cognitive processing, and visual perception were associated with FDG-PET resilience signature and PIB ratio. After adjusting for vascular risk factors, these relationships remained significant, and the effect sizes increased. DNS-br planning was only associated with FDG-PET resilience signature in our unadjusted and adjusted models. DNS-br prospective memory, eye movement, spatial memory and speech and articulation were not associated with any of the traditional brain health markers in the unadjusted model. However, in our adjusted model, DNS-br spatial memory was now significantly associated with FDG-PET resilience signature, PIB ratio, and cerebral white matter volume. DNS-br eye movement was associated with FDG-PET resilience signature, cerebral gray matter volume, and cerebral white matter volume. DNS-br prospective memory was associated with FDG-PET resilience signature and PIB ratio. DNS-br speech and articulation was not associated with any of the brain health markers.

Overall, these granular analyses highlight an interesting pattern where digital biomarker clusters that measure higher executive cognitive functioning were associated with established markers of dementia in both the adjusted and unadjusted models. Conversely, less executive cognitive functioning like speech and articulation—linked to frontal lobe areas such as Wernicke's and Broca's area (29)—were not significantly associated with established biomarkers of dementia in either model. A possible explanation for this finding is that since the DNS-br biomarkers for speech and articulation are not capturing language specific cognitive tasks, but rather a set of non-language specific features, then it is unlikely that they will be related to established biomarkers of dementia. In another interesting finding, spatial memory—linked to hippocampal function—was only associated with established biological dementia outcomes in the adjusted model. It is likely that the association between spatial memory and established dementia biomarkers may be confounded by vascular risk factors. Previous research indicates that cardiovascular risk is correlated with hippocampal structure and function, such as cortical vasoreactivity to hypercapnia (excessive carbon dioxide in blood due to inadequate respiration. Additionally, disrupted functional connectivity observed at those profiles is significantly increasing those associations (30).

### Difference between digital biomarkers and traditional biological predictors of global cognitive impairment

In Table 5, we investigated which factors predicted global cognitive impairment in the 80+ participants. As hypothesized, education, DNS-br, and ^18^F-fluorodeoxyglucose positron emission tomography (FDG-PET) resilience biomarkers were negatively associated with global cognitive impairment. While, sex, APOE4 status and AV45-PET ratio were positively associated with global cognitive impairment. Of note, our digital neural-signature of brain resilience (DNS-br) was as predictive of global cognitive impairment as FDG-PET resilience biomarker, which is characterized by uptake in the bilateral anterior cingulate cortex and anterior temporal pole (the anterior end of the temporal lobe in the middle of cranial fossa) among cognitively stable 80+ (the resilience signature). Such findings are promising because digital biomarkers are less invasive for patients, more accessible, cheaper, and facilitate repeated and longitudinal use as compared to traditional imaging exams, thus increasing likelihood of early screening for cognitive impairment and Alzheimer's dementia.

AV45-PET ratio, a proxy for cerebral blood flow in Alzheimer's and perfusion, was the strongest predictor of global cognitive impairment. This finding fills a critical gap in the literature about structural predictors of AD, as previous studies have shown that AV45-PET may be less sensitive to reflect AD severity compared FDG-PET, which may explain FDG-PET was significantly associated and AV45 was not (31). Other studies have shown that FDG-PET is not a true reflection of Aβ load (32) but instead a proxy for prodromal MCI due to Alzheimer's disease, frontotemporal lobar degeneration, and prodromal dementia with Lewy bodies in mild cognitive impairment subjects (33). Since FDG-PET did not significantly predict global cognitive impairment, we were unable to determine the nature and speed of transition from MCI to AD dementia (34). This finding raises a critical issue with reliance on biological and structural markers of dementia as they are less sensitive in identifying prodromal MCI, neurodegeneration, or dementia. However, our digital neurosignature based on previous and current findings is more sensitive to identifying prodromal disease (26).

### Predictors of cognitive change

Sex and AV45-PET status were the only predictors of cognitive change, while the interaction between sex and time, DNS-br and time, FDG-PET resilience marker and time, and AV45-PET status and time significantly predicted changes in cognition. Our findings suggest that men have greater levels of cognitive change, while individuals with greater levels of AV45-PET, a proxy for cerebral blood flow, perfusion, and amyloid burden, had lower changes in cognition. Results also suggest that over time, men had less changes in cognition, markers of brain resilience (DNS-br and FDG-PET) were associated with less cognitive changes, highlighting their protective effect in preserving cognitive functioning. Conversely, AV45-PET status over time was positively associated with changes in cognition suggesting that increases in amyloid burden may lead to worsening cognitive functioning.

### Predictors of brain resilience

Based on results presented in Table 7, two unique risk profiles emerge. In the cognitively stable group, education, history of hypertension, smoking, and atrial fibrillation were predictive of unique patterns in the digital neurosignature biomarker of brain resilience. While in the cognitively impaired group, age, education, history of hypertension, diabetes, and atrial fibrillation were predictive of different patterns in the same digital neurosignature biomarker of brain resilience. Despite the unique risk profiles, evidence shows that regardless of cognitive status (stable or impaired), vascular risk factors, such as hypertension, diabetes, smoking and atrial fibrillation, are negatively associated with brain resilience, suggesting they lower brain resilience. Adverse effects of vascular risk factors on brain resilience and brain health may be driven and mediated by resultant brain injury such as cerebrovascular disease like stroke and small vessel disease like white matter hyperintensity lesions (35). If these risk factors are left untreated blood supply to the brain is reduced affecting brain functioning, metabolism, and structure, leading to atrophy in overall brain size, white and gray matter tissue (36).

Conversely, demographic factors, like age and education, affected brain resilience differently based on cognitive status. In cognitively impaired older adults, age was positively associated, while education was negatively associated with brain resilience. In cognitively stable older adults, education was positively associated with brain resilience. These conflicting results highlight differential functions of education on brain health, where in stable older adults (37, 38), it serves as a protective factor (which is consistent with previous work), while in cognitively impaired older adults, it has the opposite effect on brain health. More years of education alone in this group did not confer greater brain resilience but instead less. While this finding contradicts extant literature on the protective role of education in brain health (39), it is consistent with recent observations, such as the Religious Orders Study (40), on cognitive reserve and highlights the grim reality that in older adults who are predisposed to cognitive impairment by other risk factors, education alone loses its protective function. Our finding that age was positively associated with brain resilience in the cognitively impaired group should be interpreted with caution as it is likely that individuals who live to 80+ years may have innate brain resilience.

### The clinical and public health implications of digital health technology

Altoida can serve as a screening, predictive, preventive, and symptom monitoring and management tool. As a screening tool, Altoida can identify individuals who may be at risk for mild cognitive impairment and dementia (specifically AD). As a predictive tool, Altoida can detect possible cognitive decline years in advance. Its predictive value highlights its preventive value, where clinicians could use Altoida to start early treatments for dementia (behavioral or pharmacological) to slow cognitive decline. Lastly, Altoida can also be used to monitor progression of cognitive decline and help clinicians better manage patients symptoms. Altoida has demonstrated excellent intra-individual longitudinal changes in cognition, it can also be used as an instrument that predicts and detects transitions in cognitive status. Specifically, a DHT, like Altoida, can be used to detect whether someone will transition from normal cognitive function to mild cognitive impairment and from mild cognitive impairment to dementia/AD (14).

Our findings add to the growing body of literature on the use of wearable and “nearable” technology sensors, surveys, games, and computer mouse movements as digital biomarkers to infer cognitive status. Altoida can be used in conjunction with wearables and nearables to longitudinally monitor cognitive status and sensitively identify slight, ephemeral, or permanent changes in cognition. To move the field of digital biomarkers in brain health forward and to increase its clinical and public health value in addressing the burden of dementia, digital health technologies, like Altoida, should be nested in home-based digital technologies to improve early detection of cognitive decline, identify which cognitive domains are affected in real-life settings, and study precise impact treatments on specific cognitive functions.

## Limitations and conclusion

While our study offers several critical insights about the benefit of digital biomarkers of dementia, these findings should be interpreted cautiously in light of a few methodological limitations. First, discrepancy in sample size between cognitively impaired and stable groups precludes us from making generalizable claims from our comparative analyses. Second, it is likely that the use of a digital technology to conduct cognitive tests may affect the individual's performance, which may be confounded by technophobia and low motivation. Third, the study sample was representative and as such were unable to perform subgroup analyses across gender and racial groups, both of which have well-evidenced differential outcomes in cognitive decline and dementia. Fourth, our study sample focused on older adults and thus we were unable to include earlier age groups to assess whether DNS-br was predictive of global cognitive function in earlier age groups. Despite these limitations, our findings make significant contributions to the literature to advance and enhance our screening and diagnostic toolbox for cognitive decline and dementia *via* digital biomarkers.

## Data availability statement

The raw data supporting the conclusions of this article will be made available by the authors, without undue reservation.

## Ethics statement

The studies involving human participants were reviewed and approved by New England Institutional Review Board in San Diego. The patients/participants provided their written informed consent to participate in this study.

## Author contributions

AS and IT conceptualized the study, oversaw all aspects of data analysis and interpretation, contributed to the development of the scientific arguments, as well as the discussion, and reviewed/edited the manuscript. RH and IT processed and analyzed the data and contributed to data interpretation. RH analyzed the data and prepared tables and figures. AS, FR, MP-V, GJ-L, and IT helped to develop the scientific arguments and contributed to data interpretation. All authors contributed to the article and approved the submitted version.

## Funding

AS's effort was supported by the National Institutes of Health (NIH): K01HL135452, R01HL152453, and P30AG043073. GJ-L was supported by NIH funding: R01HL142066, R01HL095799, and RO1MD004113.

## Conflict of interest

IT was receiving reimbursements, fees, funding, or salary from Altoida Inc., that holds or has applied for patents relating to the content of the manuscript. The remaining authors declare that the research was conducted in the absence of any commercial or financial relationships that could be construed as a potential conflict of interest.

## Publisher's note

All claims expressed in this article are solely those of the authors and do not necessarily represent those of their affiliated organizations, or those of the publisher, the editors and the reviewers. Any product that may be evaluated in this article, or claim that may be made by its manufacturer, is not guaranteed or endorsed by the publisher.

## References

[1] Alzheimer's disease facts and figures. Alzheimers Dement. (2021) 17:327–406. 10.1002/alz.12328 33756057

[2] Leuzy A Chiotis K Lemoine L Gillberg P-G Almkvist O Rodriguez-Vieitez E. Tau PET imaging in neurodegenerative tauopathies—still a challenge. Mol Psychiatry. (2019) 24:1112–34. 10.1038/s41380-018-0342-8 30635637 PMC6756230

[3] Winblad B Amouyel P Andrieu S Ballard C Brayne C Brodaty H. Defeating Alzheimer's disease and other dementias: a priority for European science and society. Lancet Neurol. (2016) 15:455–532. 10.1016/S1474-4422(16)00062-4 26987701

[4] Anblagan D Hernández MCV Ritchie SJ Aribisala BS Royle NA Hamilton IF. Coupled changes in hippocampal structure and cognitive ability in later life. Brain Behav. (2018) 8:e00838. 10.1002/brb3.838 29484252 PMC5822578

[5] Metzler-Baddeley C Jones DK Belaroussi B Aggleton JP O'Sullivan MJ. Frontotemporal connections in episodic memory and aging: a diffusion MRI tractography study. J Neurosci. (2011) 31:13236–45 10.1523/JNEUROSCI.2317-11.2011 PMC6623273 21917806

[6] MacKinnon DP Pirlott AG. Statistical approaches for enhancing causal interpretation of the M to Y relation in mediation analysis. Pers Soc Psychol Rev. (2015) 19:30–43. 10.1177/1088868314542878 25063043 PMC4835342

[7] Raymond YL William JJ. Effect of cognitive reserve markers on alzheimer pathologic progression. Alzheimer Dis Assoc Disord. (2013) 27:343–50. 10.1097/WAD.0b013e3182900b2b 23552443 PMC3745532

[8] Adams JN Lockhart SN Li L Jagust WJ. Relationships between tau and glucose metabolism reflect alzheimer's disease pathology in cognitively normal older adults. Cereb Cortex. (2019) 29:1997–2009. 10.1093/cercor/bhy078 29912295 PMC6458898

[9] Arnold SE Louneva N Cao K Wang L-S Han L-Y Wolk DA. Cellular, synaptic, and biochemical features of resilient cognition in Alzheimer's disease. Neurobiol Aging. (2013) 34:157–68. 10.1016/j.neurobiolaging.2012.03.004 22554416 PMC3478410

[10] Arenaza-Urquijo EM Przybelski SA Lesnick TL Graff-Radford J Machulda MM Knopman DS. The metabolic brain signature of cognitive resilience in the 80+: beyond Alzheimer pathologies. Brain. (2019) 142:1134–47. 10.1093/brain/awz037 30851100 PMC6439329

[11] Metzler-Baddeley C Mole JP Sims R Fasano F Evans J Jones DK. Fornix white matter glia damage causes hippocampal gray matter damage during age-dependent limbic decline. Sci Rep. (2019) 9:1060. 10.1038/s41598-018-37658-5 30705365 PMC6355929

[12] Muurling M de Boer C Kozak R Religa D Koychev I Verheij H. Remote monitoring technologies in Alzheimer's disease: design of the RADAR-AD study. Alz Res Therapy. (2021) 13:89. 10.1186/s13195-021-00825-4 33892789 PMC8063580

[13] Coravos A Khozin S Mandl KD. Developing and adopting safe and effective digital biomarkers to improve patient outcomes. NPJ Digit Med. (2019) 2:14. 10.1038/s41746-019-0090-4 30868107 PMC6411051

[14] Meier IB Buegler M Harms R Seixas A Çöltekin A Tarnanas I. Using a digital neuro signature to measure longitudinal individual-level change in Alzheimer's disease: the Altoida large cohort study. NPJ Digit Med. (2021) 4:101. 10.1038/s41746-021-00470-z 34168269 PMC8225898

[15] van den Brink W Bloem R Ananth A Kanagasabapathi T Amelink A Bouwman J. and Wopereis S. Digital resilience biomarkers for personalized health maintenance and disease prevention. Front Digit Health. (2021) 2:614670. 10.3389/fdgth.2020.614670 34713076 PMC8521930

[16] Biogen to Launch Pioneering Study to Develop Digital Biomarkers of Cognitive Health Using Apple Watch and iPhone. News release (2021). https://investors.biogen.com/news-releases/news-release-details/biogen-launch-pioneering-study-develop-digital-biomarkers (accessed January 12, 2021).

[17] Gold M Amatniek J Carrillo MC Cedarbaum JM Hendrix JA Miller BB. Digital technologies as biomarkers, clinical outcomes assessment, and recruitment tools in Alzheimer's disease clinical trials. Alzheimers Dement. (2018) 4:234–42. 10.1016/j.trci.2018.04.003 29955666 PMC6021547

[18] Babrak LM Menetski J Rebhan M Nisato G Zinggeler M Brasier N. Traditional and digital biomarkers: two worlds apart? Digit Biomark. (2019) 3:92–102. 10.1159/000502000 32095769 PMC7015353

[19] U.S. Food and Drug Administration. Digital Health Technologies for Remote Data Acquisition in Clinical In. (2021). Available online at: < https://www.fda.gov/media/155022/download> (accessed December 30, 2021).

[20] Jack CRJr Bennett DA Blennow K Carrillo MC Dunn B Haeberlein SB. NIA-AA research framework: toward a biological definition of Alzheimer's disease. Alzheimers Dement. (2018) 14:535–62. 10.1016/j.jalz.2018.02.018 29653606 PMC5958625

[21] Lim J Dinges DF. A meta-analysis of the impact of short-term sleep deprivation on cognitive variables. Psychol Bull. (2010) 136:375–89. 10.1037/a0018883 20438143 PMC3290659

[22] Musiek ES Xiong DD Holtzman DM. Sleep, circadian rhythms, and the pathogenesis of Alzheimer disease. Exp Mol Med. (2015) 47:e148. 10.1038/emm.2014.121 25766617 PMC4351409

[23] Petersen RC Wiste HJ Weigand SD Fields JA Geda YE Graff-Radford J. NIA-AA Alzheimer's Disease Framework: Clinical Characterization of Stages. Ann Neurol. (2021) 89:1145–56. 10.1002/ana.26071 33772866 PMC8131266

[24] Landau SM Horng A Fero A Jagust WJ Alzheimer's Disease Neuroimaging Initiative. Amyloid negativity in patients with clinically diagnosed Alzheimer disease and MCI. Neurology. (2016) 86:1377–85. 10.1212/WNL.0000000000002576 26968515 PMC4831042

[25] Sanborn V Preis SR Ang A Devine S Mez J DeCarli C. Association between leptin, cognition, and structural brain measures among “early” middle-aged adults: results from the framingham heart study third generation cohort. J Alzheimers Dis. (2020) 77:1279–89. 10.3233/JAD-191247 32831199 PMC7923949

[26] Buegler M Harms R Balasa M Meier IB Exarchos T Rai L. Digital biomarker-based individualized prognosis for people at risk of dementia. Alzheimers Dement. (2020) 12:e12073. 10.1002/dad2.12073 32832589 PMC7437401

[27] Stavropoulos TG Lazarou I Diaz A Gove D Georges J Manyakov NV. Wearable devices for assessing function in Alzheimer's disease: a European public involvement activity about the features and preferences of patients and caregivers. Front Aging Neurosci. (2021) 13:643135. 10.3389/fnagi.2021.643135 33912025 PMC8072390

[28] Lundberg SM Erion G Chen H DeGrave A Prutkin JM Nair B. From local explanations to global understanding with explainable AI for trees. Nat Mach Intell. (2020) 2:56–67. 10.1038/s42256-019-0138-9 32607472 PMC7326367

[29] Pilly PK Grossberg S. How do spatial learning and memory occur in the brain? coordinated learning of entorhinal grid cells and hippocampal place cells. J Cogn Neurosci. (2012) 24:1031–54. 10.1162/jocn_a_00200 22288394

[30] Sendi MSE Zendehrouh E Fu Z Liu J Du Y Mormino E. Disrupted dynamic functional network connectivity among cognitive control networks in the progression of Alzheimer's disease. Brain Connect. (2021) 11:115–62. 10.1089/brain.2020.0847 34102870 PMC10442683

[31] Ottoy J Verhaeghe J Niemantsverdriet E De Roeck E Ceyssens S Van Broeckhoven C. 18F-FDG PET, the early phases and the delivery rate of 18F-AV45 PET as proxies of cerebral blood flow in Alzheimer's disease: validation against 15O-H2O PET. Alzheimer's Dement. (2019) 15:1172–82. 10.1016/j.jalz.2019.05.010 31405824

[32] Ottoy J Verhaeghe J Niemantsverdriet E Wyffels L Somers C De Roeck E. Validation of the semiquantitative static SUVR method for 18F-AV45 PET by pharmacokinetic modeling with an arterial input function. J Nucl Med. (2017) 58:1483–9. 10.2967/jnumed.116.184481 28336779

[33] Arbizu J Festari C Altomare D Walker Z Bouwman F Rivolta J. Clinical utility of FDG-PET for the clinical diagnosis in MCI. Eur J Nucl Med Mol Imaging. (2018) 45:1497–508. 10.1007/s00259-018-4039-7 29704037

[34] Morbelli S Bauckneht M Arnaldi D Picco A Pardini M Brugnolo A. 18F-FDG PET diagnostic and prognostic patterns do not overlap in Alzheimer's disease (AD) patients at the mild cognitive impairment (MCI) stage. Eur J Nucl Med Mol Imaging. (2017) 44:2073–83. 10.1007/s00259-017-3790-5 28785843

[35] Mathur RK. Role of diabetes, hypertension, and cigarette smoking on atherosclerosis. J Cardiovasc Dis Res. (2010) 1:64–8. 10.4103/0975-3583.64436 20877688 PMC2945206

[36] Cox SR Lyall DM Ritchie SJ Bastin ME Harris MA Buchanan CR. Associations between vascular risk factors and brain MRI indices in UK Biobank. Eur Heart J. (2019) 40:2290–300. 10.1093/eurheartj/ehz100 30854560 PMC6642726

[37] Giacomucci G Mazzeo S Padiglioni S Bagnoli S Belloni L Ferrari C. Gender differences in cognitive reserve: implication for subjective cognitive decline in women. Neurol Sci. (2022) 43:2499–508. 10.1007/s10072-021-05644-x 34625855 PMC8918152

[38] Morris TP Ai M Chaddock-Heyman L McAuley E Hillman CH Kramer AF. Relationships between enriching early life experiences and cognitive function later in life are mediated by educational attainment. J Cogn Enhanc. (2021) 5:449–58. 10.1007/s41465-021-00208-5 35005424 PMC8741175

[39] van Arendonk J Yilmaz P Steketee R Zijlmans JL Lamballais S Niessen WJ. Resistance to developing brain pathology due to vascular risk factors: the role of educational attainment. Neurobiol Aging. (2021) 106:197–206. 10.1016/j.neurobiolaging.2021.06.006 34298318

[40] Wilson RS Yu L Lamar M Schneider JA Boyle PA Bennett DA. Education and cognitive reserve in old age. Neurology. (2019) 92:e1041–50. 10.1212/WNL.0000000000007036 30728309 PMC6442015

